# APRIL promotes non-small cell lung cancer growth and metastasis by targeting ERK1/2 signaling

**DOI:** 10.18632/oncotarget.22672

**Published:** 2017-11-27

**Authors:** Hengli Dou, Zhaohua Yan, Meng Zhang, Xiaoxin Xu

**Affiliations:** ^1^ Department of Neurosurgery, The Fourth Hospital of Jinan, Jinan 250013, Shandong, China

**Keywords:** lung cancer, APRIL, BCMA, TACI, proliferation

## Abstract

Non-small-cell lung cancer (NSCLC) is the major subtype of lung cancer, which is the most common cause of cancer-related mortality in the world. It is a complex disease involving multiple genetic alterations. As a cytokine belonging to the Tumor Necrosis Factor-α (TNF- α) family, the - a proliferation-inducing ligand (APRIL) expression and its signaling have been studied in many human solid tumor types, but the data on APRIL signaling in NSCLC are lacking. The aim of this study was to evaluate the APRIL expression and investigate its signaling in NSCLC. The expression of APRIL and its receptors, B cell maturation antigen (BCMA) and transmembrane activator and calcium-modulatorand cyclophilin ligand interactor (TACI), was analyzed by using immunohistochemistry in NSCLC samples. Quantitative RT-PCR was performed to evaluate mRNA expression of APRIL, BCMA and TACI in human lung adenocarcinoma cell lines A549, H1299, and H1650. Cell proliferation was measured by using the cell proliferation and cytotoxicity assay kit 8 (CCK8) assay, cell migration by using wound healing assay, and cell invasion by using transwall assay. The protein level of APRIL, BCMA and TAC, and the activation of extracellular regulated protein kinases 1/2 (ERK1/2) signaling, were determined by western blot. Our results indicated, APRIL and its receptors BCMA and TACI, were overexpressed in most of human NSCLC samples and cell lines; APRIL promoted tumor proliferation, migration and metastasis in A549 and H1299 cells via BCMA and TACI. Furthermore, ERK1/2 activation was involved in APRIL signaling through TACI but not BCMA in A549 and H1299 cells. APRIL might serve as a potential prognostic biomarker for NSCLC, and APRIL related signaling pathway could be a therapeutic target for NSCLC.

## INTRODUCTION

Non-small cell lung cancers (NSCLCs) are the predominant forms of lung cancers and account for the majority of cancer deaths worldwide [1]. There are two major histological types of NSCLCs: adenocarcinoma (AD) and squamous cell carcinoma (SC) [1]. Great progresses have been made in the understanding of the pathogenesis and treatment approaches of NSCLCs in the last decade [2–6]. However, despite significant improvements in surgery, chemotherapy and radiotherapy, the prognosis of lung cancer remains poor, with only slightly more than 15% of patients alive 5 years after diagnosis [7], mainly because that most patients have been already in the advanced NSCLC when they were diagnosed. Currently, major means to diagnose NSCLC is mainly X-ray, CT, and sputum cytology etc. All the methods have their own limitations, in particular, the failure to determine precisely malignance stages [8]. Therefore, there is an urgent need to find new molecular biomarkers for early diagnosis of NSCLC and effective therapeutic targets to improve the treatment of NSCLC.

Cytokines are important molecules in the development and progression of NSCLC [9, 10]. They can be secreted by tumor cells or surrounding cells, and construct complex communication networks to promote cancer cell proliferation and survival, as well as invasion and metastasis [9, 10]. APRIL (a proliferation-inducing ligand) is a cytokine belonging to the TNF family, originally named for its ability to stimulate tumor cell proliferation *in vitro* [11]. APRIL is involved in the regulation of B-cell homeostasis by promoting peripheral B-cell survival, maturation, and differentiation. APRIL binds to two known receptors: transmembrane activator and calcium-modulator and cyclophilin ligand interactor (TACI), and B cell maturation antigen (BCMA) [12, 13]. TACI serves as a high affinity receptor for APRIL, while BCMA binds APRIL only weakly. TACI is known to mediate extracellular signal-regulated kinase (ERK) 1/2 mitogen-activated protein (MAP) kinases (ERK1/2-MAPK) signaling in B cells and macrophage [14, 15]. Akt and JNK pathways are involved in the regulation of BCMA in multiple myeloma [16]. The association between APRIL and cancers has been studied in leukemia and lymphoma, after the initial description of APRIL receptors in B cells [17]. It is also reported that APRIL transgenic mice develop lymphoid tumors [18]. Overexpression of APRIL has also been reported in many human solid tumor types, such as hepatocellular carcinoma [19], glioblastoma [20], pancreatic cell lines [21], colon carcinoma [22], and breast cancer [23, 24]. APRIL is overexpressed in breast tissue lesions and cancer cell lines, but is associated mainly with the stroma and non-malignant structures [23]. Recently, upregulation of APRIL at the transcript and protein level in NSCLC cells, stromal fibroblast, and chronic obstructive pulmonary disease (COPD) patients with NSCLC have been reported, but the data on APRIL signaling in NSCLC are very limited. Its involvement in lung tumorigenesis and metastasis, and the underlying molecular mechanisms are rarely known. In the present study, we sought to address roles of APRIL and its signaling in NSCLC.

Here, we found that APRIL, BCMA and TACI were overexpressed in human NSCLC cell lines and primary tumor samples. Using cell lines *in vitro*, we confirmed that ectopic APRIL promoted cancer proliferation, and BCMA or TACI silencing reduced migration and invasion of A549 and H1299 cells. Furthermore, we found that APRIL signaled through extracellular signal-regulated kinase (ERK) 1/2 mitogen-activated protein (MAP) kinases. Most interesting finding was that the ERK signaling was medicated by binding the TACI receptor but not BCMA. Our results suggested an association between this APRIL signaling pathway and NSCLC tumor growth.

## RESULTS

### APRIL, BCMA and TACI are overexpressed in human NSCLC samples

**Figure 1:**
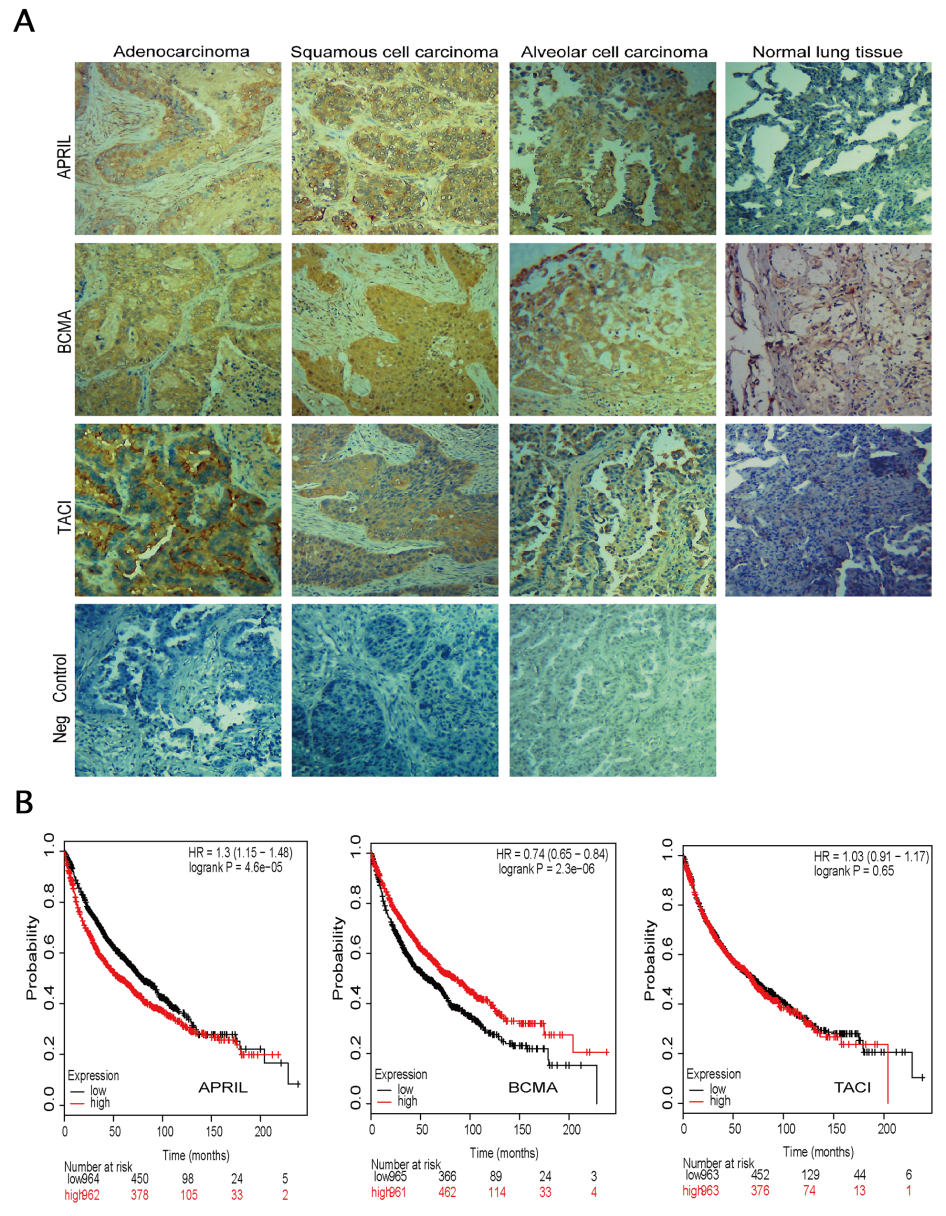
Expression of APRIL, BCMA and TACI in lung specimens **(A)** Immunohistochemistry in cancerous tissue samples, showing abnormal expression of APRIL, BCMA, TACI and negative control (same tissue but without primary antibody). Tissue sections from 3 subtypes of NSCLC were stained with these three antibodies respectively or without primary antibody. Brown staining indicates immunopositive cells. **(B)** Survival curves were plotted for NSCLC patients.

In order to investigate the role of APRIL and its receptors in NSCLC, we firstly evaluated the expression levels of APRIL, BCMA and TACI by using immunohistochemistry in tissue sections from human NSCLC samples tissue micro array (Figure 1A). The TMA consisted of three types of tumors, including adenocarcinoma (AD), squamous cell carcinoma (SC) and alveolar cell carcinoma. Normal tissues were also included as control. In total, 62 men and 18 women formed the group, and the mean age was 53.4 years old (range, 18–90 years old). The characteristics and pathological classifications of primary tissue samples were shown in Table 1. Positive tumors include adenocarcinomas, squamous cell carcinomas and alveolar cell carcinoma. The positive staining of APRIL was found in three different subtypes of NSCLC (Figure 1A). Overexpression of APRIL was observed in 24 out of 48 lung cancers, whereas there was only one case of 7 normal lung tissues with APRIL overexpression (*P*<0.05) (Table 1 and Figure 1A). Further analyses indicated that the high expression level was not restricted to any specific subtypes of lung cancers. Also there was no correlation between APRIL with other factors such as age, gender, tumor stages, or pathologic types (Table 1). Expression of BCMA and TACI in lung cancer specimens was confirmed by immunohistochemistry as well. Immunohistochemistry assay also revealed the overexpression of BCMA and TACI in NSCLC tissues (Figure 1A). The expression of BMCA was elevated in 31 out of 48 NSCLC samples and only one of seven samples of normal lung tissue (*P*<0.01) (Table 2); 32 out of 72 NSCLC samples showed elevated TACI expression, in contrast to only 7 out of 74 healthy lung tissue samples (*P*<0.01) (Table 3). These data confirmed that the overexpression of APRIL, BCMA and TACI occurred in lung cancers, but were not restricted to any specific subtypes.

**Table 1:** The relationship between APRIL expression level and clinicopathologic feature

Clinicopathologic feature	Cases (n)	APRIL		P value
		Low-expression (n=)	High-expression (n=)	
Age				
≤60	32	17	15	0.5403
>60	16	7	9	
Sex				
male	31	18	13	0.1313
female	17	6	11	
Grades				
I-II	19	7	12	0.2333
III	10	6	14	
Pathological Classification				
adenocarcinoma	8	3	5	0.3865
squamous cell carcinoma	16	9	7	
Lymph-node metastasis				
With	16	10	6	0.3638
without	25	12	13	

**Table 2:** The relationship between BCMA expression level and clinicopathologic feature

Clinicopathologic feature	Cases (n)	BCMA		P value
		Low-expression (n=)	High-expression (n=)	
Age				
≤60	32	14	18	0.0878
>60	16	3	13	
Sex				
male	31	15	16	0.0921
female	17	3	14	
Grades				
I-II	19	5	14	0.7056
III	10	2	8	
Pathological Classification				
adenocarcinoma	10	1	9	0.3451
squamous cell carcinoma	16	4	12	
Lymph-node metastasis				
with	16	4	12	0.942
without	25	6	19	

**Table 3:** The relationship between TACI expression level and clinicopathologic feature

Clinicopathologic feature	Cases (n)	TACI		P value
		Low-expression (n=)	High-expression (n=)	
Age				
≤60	34	21	13	0.3159
>60	38	19	19	
Sex				
male	46	23	23	0.7656
female	28	15	13	
Grades				
I-II	45	23	22	0.6106
III	10	6	4	
Pathological Classification				
adenocarcinoma	29	18	11	0.0271
squamous cell carcinoma	30	10	20	

### Patients with high APRIL level show poorer overall survival in NSCLC

We evaluated the prognosis value of APRIL, BCMA and TACI on Kaplan-Meier plotter database (www.kmplot.com). Kaplan-Meier plotter (KM plotter) provides prognostic information and mRNA mapping of four type cancer patients, including lung, breast, gastric and ovarian cancer from GEO (Affymetrix microarrays only), EGA and TCGA. This online database could assess the prognosis value of gene or gene combination though analyzing the Affymetrix IDs of which on the dataset. The valid Affymetrix IDs of APRIL, BCMA and TACI are 201306_s_at, 206641_at and 207641_at, respectively. As shown in Figure 1B, the survival curve demonstrated a significant correlation between patients with low APRIL expression level and a good prognosis. The high expression of BCMA was associated with better overall survival (OS), while, TACI expression level was observed to be not significantly associated with prognosis in NSCLC. These results suggested a potential prognosis value of APRIL in NSCLC (Figure 1B).

### NSCLC cell lines express APRIL and its receptors BCMA and TACI

**Figure 2:**
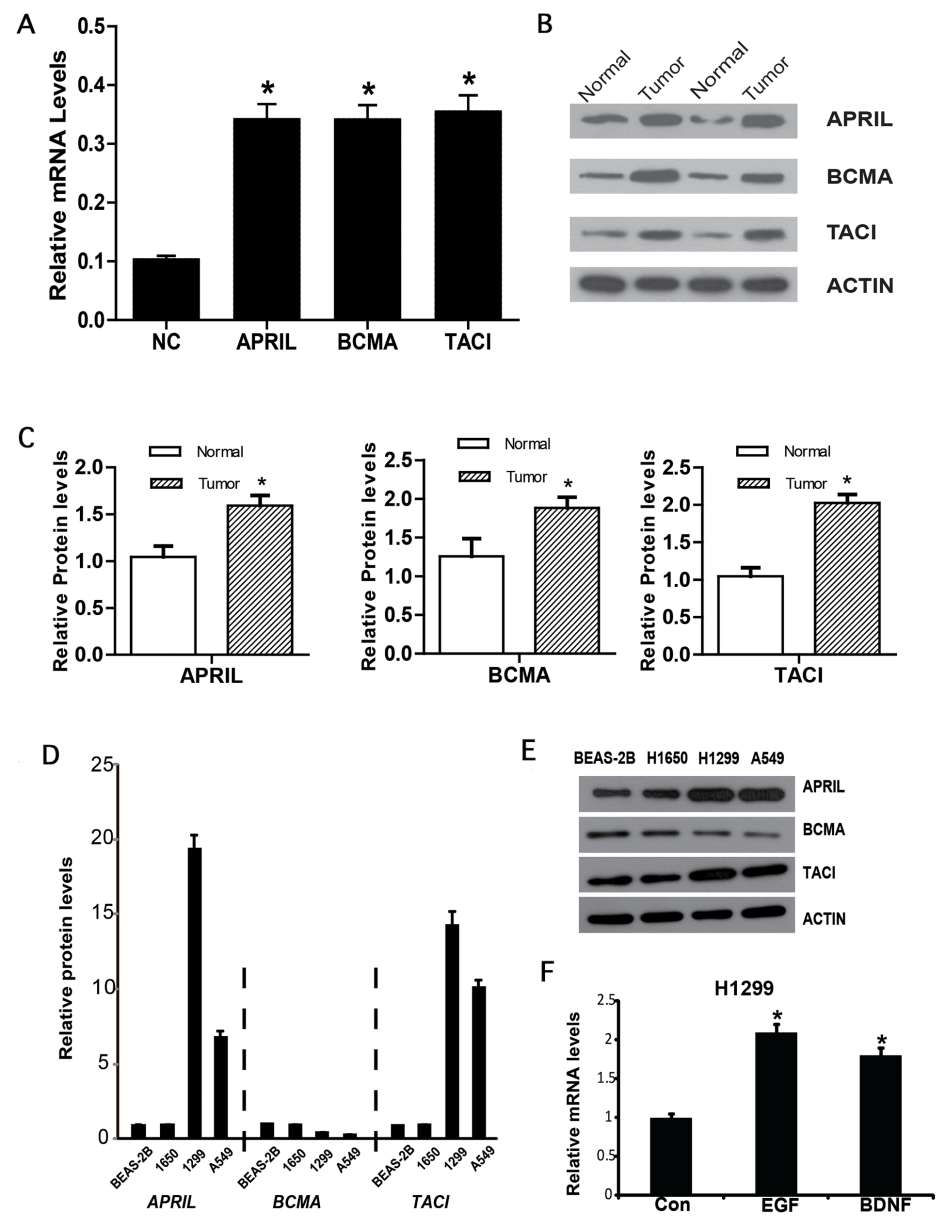
APRIL, BCMA and TACI expression in lung cancer **(A)** Relative mRNA expression of APRIL, BCMA and TACI in NSCLC tissues. **(B, C)** Western blot results of APRIL and its reporters in NSCLC tissues. **(D)** Relative *APRIL*, *BCMA* and *TACI* mRNA expression in three lung cancer cell lines (A549, H1650 and H1299). **(E)** Western blot analysis of cell lysates showing protein levels of APRIL, BCMA and TACI. **(F)** A549 cells were stimulated with EGF or BDNF. qRT-PCR analysis shows *APRIL*, *BCMA* and *TACI* mRNA levels. Values were mean ± SD (n = 3 independent experiments). ^*^ stands for significant difference (*p< 0.05*)

To further confirm the expression level of APRIL, BCMA and TACI, cancer tissue and precancerous tissue of NSCLC patients were collected. Western blot and qPCR were performed to detect the gene expression on protein and mRNA level. As shown in Figure 2A-2C, the expression level of APRIL and its receptors significantly increased in cancer tissues compared with in precancerous tissues. Further, we measured APRIL expression in three human NSCLC cell lines with different features. qRT-PCR analysis of *APRIL* mRNA showed APRIL transcripts in all four cell lines. The mRNA level of *APRIL* significantly increased in human lung adenocarcinoma cell lines in comparison to human bronchial epithelial cell line BESA-2A, with a maximum nine-fold difference between BEAS-2B and H1299 (highest) (Figure 2D). We also analyzed BCMA and TACI transcripts by qRT-PCR in the four cell lines, and observed higher levels of TACI in H1299 and A549 compared with BEAS-2B. Western blots confirmed expression of APRIL, BCMA and TACI in the cell lines (Figure 2E). Next, we examined whether epidermal growth factor (EGF) or brain derived neurotrophic factor (BDNF) regulated the expression of APRIL, BCMA and TACI. As EGF and BDNF have been reported to contribute to NSCLC development [25–28]. We examined their role in APRIL expression in H1299 cells, which showed elevated APRIL expression in comparison to H1650 and A549 cells. mRNA measurement indicated that both EGF and BDNF enhanced APRIL transcription after incubation for 24h in H1299 cells (Figure 2F).

### Ectopic APRIL promotes cell proliferation in A549 and H1299 cells

**Figure 3:**
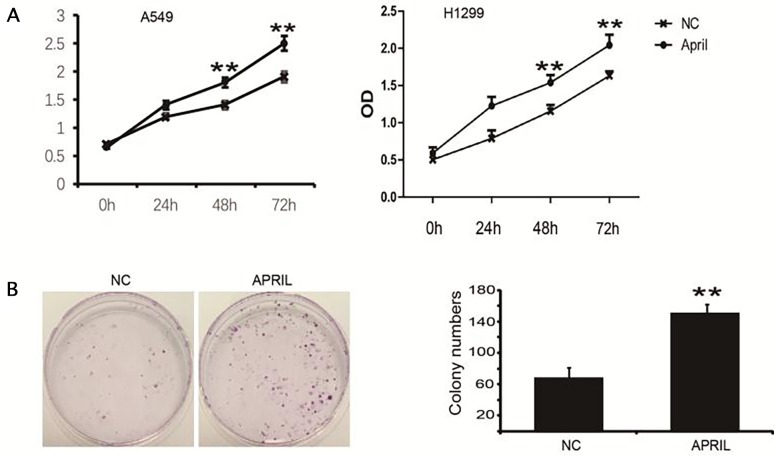
APRIL promotes cell proliferation and colony formation in H1299 cells **(A)** Proliferation of H1299 cells was markedly increased with APRIL. H1229 cells were incubated with APRIL. And, the proliferation was measured by CCK-8 kit. **(B)** H1229 cells were treated w/o APRIL, colony formation was analyzed by crystal violet staining. Sizes of H1299 cells were significantly greater after treatment with APRIL. Values were mean ± SD (n = 3 independent experiments).

To explore the role of APRIL on NSCLC, we performed the CCK-8 assay to determine its effect on the proliferation of H1299 and A549 cells. We treated cells with APRIL (200ng/ml) for indicated times. OD values at 450 nm in the APRIL group were significantly higher than those in control groups at 48 and 72 h (*P*<0.05) (Figure 3A). We also performed a colony formation assay to confirm this result. The colony-forming activity of the APRIL group was higher than that in the negative control groups (Figure 3B). These data indicated that APRIL can promote the proliferation capacity of NSCLC cells.

### APRIL promotes tumor migration and metastasis in A549 and H1299 cells through BCMA and TACI

**Figure 4:**
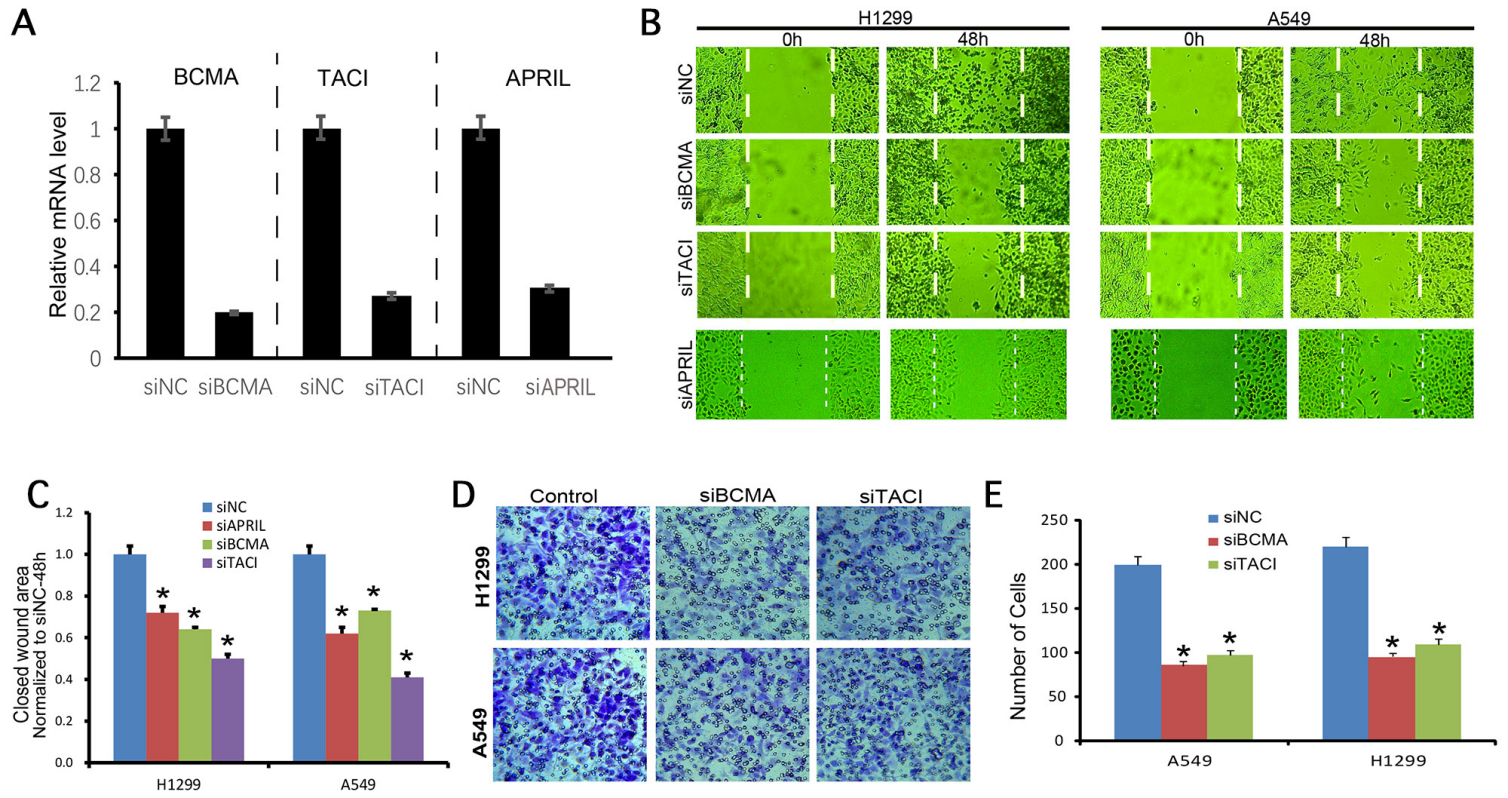
BCMA and TACI silencing decreases migration and invasion of NSCLC cells **(A)** the knockdown efficiencies of APRIL, BCMA, and TACI were detected by RT-PCR, which indicated the expression of these three genes was significantly decreased in each knockdown group. (B, C) Wound healing assay photograph **(B)** and quantification **(C)** showing that the migration ability of H1299 and A549 cells was lower in the siBCMA and siTACI groups 24 h after wounding than in the respective siNC groups (P < 0.05). (D, E) Transwell assay photograph **(D)** and quantification **(E)** showing that the invasive abilities of A549 and H1299 cells were lower in the siBCMA and siTACI group after 24 h of incubation than in both siNC groups (P < 0.05). Values are mean ± SD (n = 3 independent experiments).

A549 and H1299 cells were transfected with siRNAs against APRIL, BCMA and TACI. The qRT-PCR result showed that siRNA significantly decreased expression of APRIL, BCMA and TACI mRNA (Figure 4A). Wound healing was performed to investigate the effect of APRIL on cell migration. As shown in Figure 4B and 4C, the migratory ability in siAPRIL group was significantly decresed at 48h both in A549 and H1299 cells (*P*< 0.05). To further investigate the mechanisms by which APRIL promotes migration, we examined the effects of the siBCMA and siTACI on migration. We performed wound healing and transwall assays to investigate the effect of BCMA and TACI on the migratory and invasive potential of A549 and H1299 cells after transfection with siRNAs. Wound healing assay revealed that the migratory ability of A549 and H1299 cells in the siRNA transfection group were both significantly decreased at 48h after wounding, compared with the respective NC groups (Figure 4B and 4C, P<0.05). Cell invasion assay showed that the number of invading cells was significantly lower in the siRNA group than that in the NC control group both in A549 and H1299 cells (Figure 4D and 4E, *P*<0.05).

### APRIL activates ERK1/2 MAP kinase

**Figure 5:**
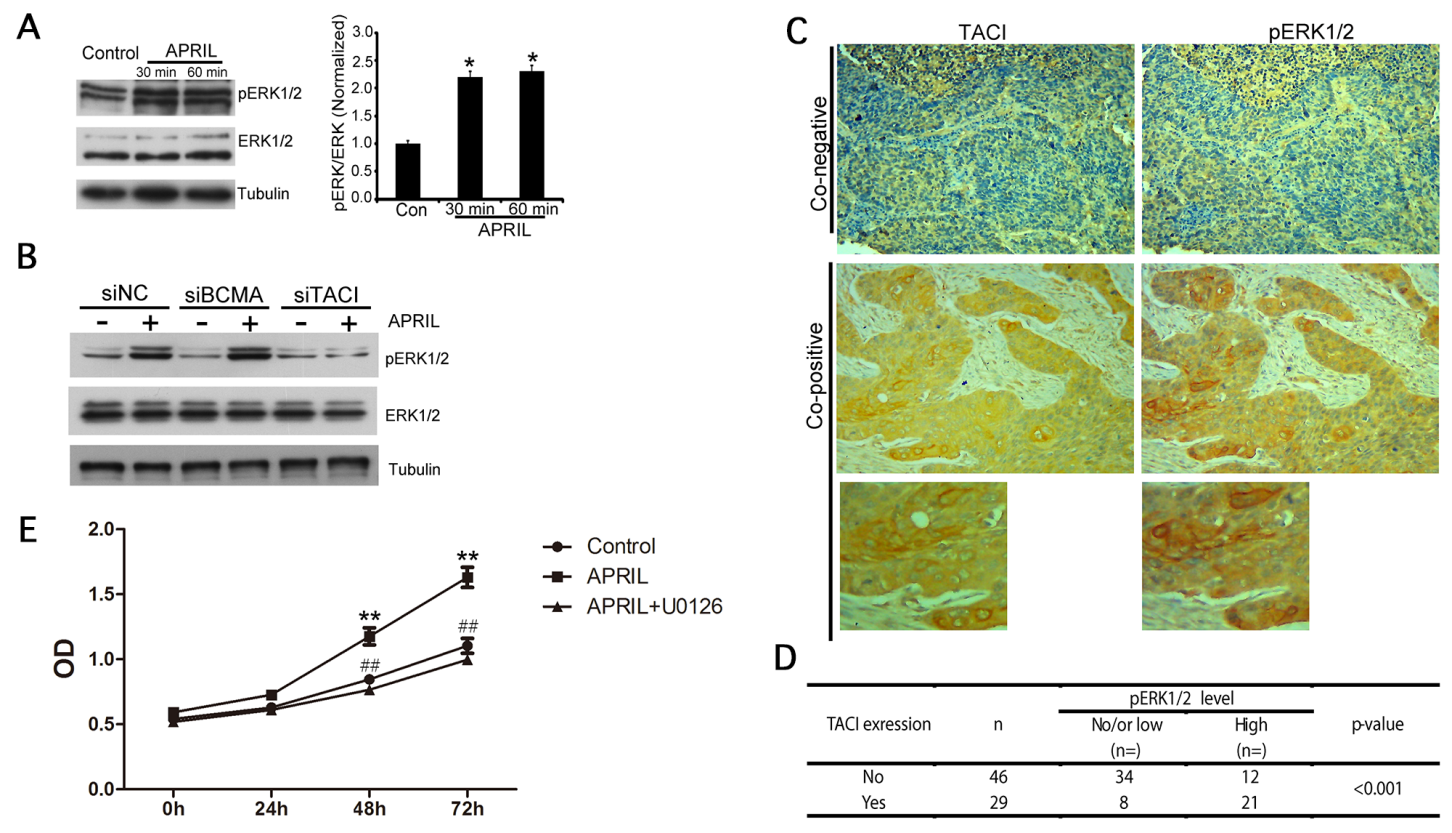
APRIL activates ERK1/2 MAP kinase **(A)** Western blot analysis of lysates from A549 cells 0 min (control), 30 min and 60 min after activation with APRIL (200 ng/ml). ERK1/2 phosphorylation was detected. Tubulin was used as a loading control. Protein levels were quantified by Image J. **(B)** ERK1/2 phosphorylation level was detected in A549 cells silencing BCMA or TACI w/o APRIL treatment. **(C, D)** Expression of TACI and phosphorylated p-ERK1/2 in NSCLC samples was detected by immunohistochemistry. The correlation between TACI and p-ERK was analyzed in Figure (D). **(E)** CCK8 assay showed that the ERK1/2 inhibitor U0126 blocked the cell response to APRIL. Values were mean ± SD (n = 3 independent experiments).

The finding that NSCLCs abnormally overexpressed APRIL, TACI and BCMA predicted that signaling through these receptors might be involved in tumor growth. We therefore measured APRIL-mediated signaling via the MAP kinase ERK1/2, which has been implicated in NSCLC tumorigenesis [29–33]. A549 and H1299 cells were treated with APRIL for 30 and 60 min, respectively. Analysis of phosphorylation levels of ERK1/2 in A549 and H1299 cells showed that APRIL rapidly induced ERK1/2 phosphorylation in 30 and 60 minutes (Figure 5A). This indicated that ERK1/2 kinase was activated by APRIL in NSCLC cells. Then, we tried to further examine whether APRIL activated ERK1/2 phosphorylation through both BCMA and TACI receptors. As shown, the p-ERK1/2 level was evaluated in A549 cells with two receptors silenced (Figure 5B). Apparently, p-ERK1/2 was significantly abolished in cells with TACI knockdown but not in cells with BCMA knockdown, suggesting that the activated ERK1/2 signaling triggered by APRIL was mediated by TACI receptor but not BCMA. Furthermore, the phosphorylation level of ERK1/2 in NSCLC tissue samples was detected by IHC (Figure 5C). We analyzed the associations between pERK1/2 staining and TACI expression in 75 cases of NSCLC samples and observed a positive association. We used single staining in the adjacent slices. In 29 samples with high TACI expression, 21 of them showed high p-ERK1/2 activation (co-positive). In contrast, in 46 samples without TACI expression, only 12 of them showed high p-ERK1/2 level (*P*< 0.001, Figure 5D). To determine whether ARPIL promoted cell proliferation by ERK1/2 activation and whether inhibition of ERK1/2 would abolish APRIL-induced cell proliferation in H1299 cells, a CCK-8 assay was performed in cells with ARPIL or with APRIL plus ERK1/2 inhibitor, U0126. U0126 is a highly selective inhibitor on both MEK1 and MEK2, a type of MAPK/ERK kinase, blocks ERK pathway. In APRIL group, the rate of cell proliferation significantly increased, while it dropped to levels of NC group in the APRIL+U0126 group. U0126 addition markedly compromised the cell proliferation promoted by APRIL, indicating that ERK1/2 inhibition did block the cell response to APRIL (Figure 5E). We also detected Wnt and PI3K signaling pathways, but no significant changes in the related proteins in the pathway were observed after APRIL treatment, indicating that the pathway was not involved in APRIL regulation (data not shown).

## DISCUSSION

Recent studies point to APRIL as a new candidate molecule to be incorporated into the arsenal of therapeutic targets against cancers [11, 19, 20, 22, 23, 34, 35]. Also, it has been proposed as a biomarker for NSCLC years ago (Sun B, et al., 2009). However, the APRIL pathway has not been explored in NSCLC samples, and the downstream signaling pathway involved in APRIL function in NSCLC was not reported also. Here, we reported that APRIL, and its receptors, BCMA and TACI, were abnormally expressed in human NSCLC cell lines and primary tumor samples. We found that APRIL was functional through ERK1/2MAP kinases by binding to TACI receptor. We confirmed in different cell lines that APRIL promoted cancer cell proliferation, migration and invasion in NSCLC. Our results suggested an association of the APRIL signaling pathway with NSCLC.

Our results provide several new insights into the mechanism and significance of APRIL-related NSCLC. First, we found that the NSCLC patients with high APRIL level showed poorer prognosis. Interestingly, the prognostic role of BCMA was opposite to that of APRIL, suggesting that BCMA may have other unknown effects or regulatory mechanisms in NSCLC. Furthermore, APRIL, BCMA and TACI were overexpressed in human NSCLC cell lines as well as in primary tumor samples. However, according to the immunohistochemistry results, there was no correlation between APRIL, BCMA and TACI with other factors such as tumor stages or pathological types. These results suggested that there may be other compensatory mechanisms in the NSCLC tissues, which required further experiments and more tissue samples to be validated. Nevertheless, APRIL and its receptor still remained a potentially clinically valuable as tumor promoting factors according to the results of present study. Over the past decade, a growing number of biomarkers have been identified for NSCLC. Our result indicated that APRIL may serve as a biomarker for their high expression rate.

Second, previous reports [5, 6] show that EGFR is overexpressed and abnormally activated in lung cancers, and that exogenous BDNF increases TrkB activation and affects cancer cell signaling [28]. APRIL is found to be regulated by p38/CREB signaling pathway. Both of EGF and TrkB are well known regulator of CREB activity. Our results showed that APRIL expression was under the control of EGF and BDNF signaling in NSCLC cells, meaning that these two factors are critical inducers of APRIL in lung cancer cells. This also provided new clues to the mechanism of EGF and BDNF signaling regulation.

Third, we found that APRIL promoted tumor proliferation, migration and metastasis in A549 and H1299 cells via BCMA andTACI. APRIL increased cell progression; BCMA and TACI silencing inhibited cell migration and invasion. These results indicated that APRIL signaling might play a role in the progression of NSCLCs by affecting cell proliferation, migration and invasion. However, wound healing and transwall assays showed both receptors played a role in migration and invasion, but only TACI can mediate p-ERK1/2 activation, indicating the p-ERK1/2 activation through TACI but not BCMA. Using a CCK-8 assay, we found that APRIL promoted cell proliferation in NSCLC cells, and that this response was lost when the cells were pretreated with the ERK1/2 inhibitor U0126. These results suggested that the ERK1/2 activation was involved in APRIL signaling in NSCLC *in vitro*. In combination with the inconsistent prognosis value of BCMA and APRIL, we predict that BCMA is not only a APRIL receptor, but may play other important roles in NSCLC and take participate in the regulation of other signaling pathways. This will also be one of the focuses of further research.

In summary, we have shown here that APRIL was overexpressed in human NSCLC samples, suggesting APRIL could serve as a biomarker for NSCLC early diagnosis. APRIL promotes tumor proliferation, migration and metastasis in A549 and H1299 cells via BCMA and TACI, but signaling downstream of ERK1/2 activation only through TACI, indicating the APRIL’s functions on tumor proliferation, migration and metastasis are not activated only by ERK1/2 signaling. The signaling mediated by BCMA receptor might also be involved in this activation and need to be further investigated. However, TACI receptor or ERK signaling pathway still could be a therapeutic target for NSCLC.

## MATERIALS AND METHODS

### Reagents and tissue samples

Recombinant human APRIL was purchased from ProSpec-TanyTechnoGene (East Brunswick, NJ, USA). Antibodies against APRIL (ab64967), BCMA (ab5972), and TACI (ab64967) were purchased from Abcam (Cambridge, MA, USA). Rabbit anti-ERK1/2, mouse anti-ERK1/2 and rabbit anti-phospho-Akt S473 antibodies were from Cell Signaling Technology (Danvers, MA, USA). Other reagents were from Sigma-Aldrich (St. Louis, MO, USA), except where specifically indicated. The lung cancer tissue microarray was obtained from Shanghai Zhuoli Biotechnology Co., Ltd. (Shanghai, China).

### Immunohistochemistry

Formalin-fixed and paraffin-embedded tissue sections (6 μm thickness) from 48 samples of NSCLC were used for immunohistochemistry with APRIL and BCMA antibodies; 74 samples of NSCLC were used for immunohistochemistry with TACI antibodies (the 48 samples used to detect APRIL and TACI was contained). In some cases, the clinical pathological information was not complete, so the total cases of grades, pathological classification and lymph-node metastasis were less than total cases. Sections were deparaffinized with xylene, rehydrated in descending concentrations of ethanol and boiled for 10 min in citrate buffer for antigen retrieval. Endogenous peroxidase activity was suppressed with 3% H_2_O_2_ for 10 min. Slides were blocked with goat serum and incubated with the primary antibodies listed above for 1 h at room temperature, then stained with ElivisionTMplus apolymer HRP (mouse/rabbit) immunohistochemistry kit (FuzhouMaixinBiotech. Co., Ltd., Fuzhou, China). For negative controls, the primary antibody was omitted. Images were taken by a NikonEclipse TE 2000-U microscope. All of the images were collected and processed by MetaMorph software (UniversalImaging Corporation, West Chester, PA, USA).

### Cell culture and transfection

The human bronchial epithelial cell line BESA-2A and human lung adenocarcinoma cell lines A549, H1650 and H1299 were obtained from the Type Culture Collection of the Chinese Academy of Sciences (Shanghai, China). Cells were cultured in Dulbecco’s modified Eagle’s medium (DMEM) (Gibco, Carlsbad, CA, USA) containing 10% fetal bovine serum (Gibco), 100 IU/mL penicillin, and 100 μg/mL streptomycin (Invitrogen, Carlsbad, CA, USA) at 37°C in a humidified, 5% CO_2_ atmosphere. Transient transfection was performed using Lipofectamine2000 (Invitrogen). SiRNAs were synthesized by RiboBio (Guangzhou, China). The target sequences for each genes were as follows: TACI, 5_-CAGCGGAGTGGAGAAGTTGAA-3_; BCMA, 5_-CAGCGGAGTGGAGAAGTTGAA-3. qRT-PCR was used to evaluate the knockdown efficiency.

### RNA isolation and quantitative real-time PCR

Total RNA of cells was extracted by using an Ultrapure RNA Kit (CWbio, Beijing China) according to the manufacturer’s protocol. Reverse transcription was performed by using a First Strand cDNA Synthesis Kit (Qiagen, Hilden, Germany), following the manufacturer’s instructions. The relative mRNA levels of APRIL, BCMA and TACI were determined by quantitative real-time PCR (qRT-PCR), using the UltraSYBR Mixture (CWbio). Quantitative RT-PCR was performed in a cycler (Light Cycler 2.0; Roche). The relative expression levels of APRIL, BCMA and TACI mRNA were calculated using the 2^-ΔΔCt^ method and normalized to GAPDH. All primers were synthesized by GeneWiz (Beijing, China) as follows: APRIL–F, 5’ CCAGGATGCTGGAGTTTATC 3’; APRIL–R,5’TGGGAGGGCATACTTCTT3’; BCMA–F, 5’CTGTTTGGGACTGAGCTAATA3’; BCMA–R, 5’AGGCCTCTCGGAAGAATAA3’; TACI–F, 5’GGGTGGCTATGAGATCCT3’; TACI–R, 5’GAACTTGCCTTGCTCCTT3’; GAPDH–F, 5’CGGAGTCAACGGATTTGGTCGTAT3’; GAPDH–R, 5’AGCCTTCTCCATGGTGGTGAAGAC3’.

### Cell counting kit-8 assay

The cell counting kit-8 (CCK-8) assay was performed following the manufacturer’s protocol (Solarbio, Beijing, China). The three experimental groups (APRIL, APRIL+U0126 and NC) of A549 and H1299 cells were seeded into 96-well plates at a density of 1×10^4^ cells/well, with three replicate wells per group. The cells were cultured for 3 days. Then 10 μl CCK-8 solution was added to each well and incubated for a further 2 h at 37°C. The final optical density (OD) was measured at a wavelength of 450 nm to estimate cell proliferation in the different groups. The experiment was repeated three independent times.

### Colony formation assay

A549 and H1299 cells were trypsinized, counted, plated in a 6-well plate at a density of 100 cells/well, and incubated at 37°C for 14 days. The culture medium was changed every 2 days. The cell colony was fixed with 4% paraformaldehyde for 10 min and stained with crystal violet for 20min. Cell colonies were counted under a light microscope and photographed using a digital camera. All samples were in triplicate.

### Wound healing assay

Cells were seeded onto 6-well plates (2×10^5^ cells/well) and cultured for 24 h. A sterile 200 μL pipette tip was used to scratch a straight line through the cell layer in each well. Cells were incubated with fresh medium (containing 2% serum) for 48 h at 37°C in a humidified, 5% CO_2_ atmosphere; and then the wound closure was quantified at 48h post-wound by measuring the remaining unmigrated area using ImageJ. Assays were performed three times.

### Transwell assay

Cell suspension (200 μL at 2×10^5^ cells/mL) was added to the upper chamber of a 24-well Transwell plate containing polycarbonate filters with 8 μm pores (Costar, Corning, NY, USA). In the lower chamber, 500 μL of DMEM containing recombinant human APRIL (200ng/ml) was added to all groups except the control. Cells were incubated for 24 h at 37°C in a humidified, 5% CO_2_ atmosphere. Three replicate wells were used per group. After incubation, the medium was removed from the upper chamber and cells in each group were scraped off with a cotton swab. The cells that had migrated to the lower surface of the membrane were fixed with 4% paraformaldehyde for 20 min and stained with 0.1% crystal violet for 5 min. Migrating cells were counted under a microscope at 200×magnification in five random fields of view. Three independent experiments were performed.

### Protein extraction and western blotting

The cell was washed with PBS and lysed with 500 μl of TNE buffer (show recipe here) on ice. Lysates were got by pipetting, then removed and centrifuged for 15 min at 12,000 rpm. The supernatant was collected and the protein concentration was measured by BCA reagents. Protein expression was measured by western blot. Briefly, equal amounts of protein (10 μg per lane) were separated by SDS-PAGE electrophoresis, and transferred to a PVDF membrane (Bio-Rad, Hercules, CA, USA). The membrane was incubated in blocking buffer for 1 h and then probed at 4°C overnight with primary antibody. They were then rinsed with TBST and incubated with HRP-conjugated secondary antibody (1:5000) for 1 h at room temperature, followed by chemiluminescent detection.

### Statistical analysis

Experimental data were presented as the mean ± SD. All statistical analysis was performed using SPSS 13.5. Student’s *t*-test and one-way analysis of variance (ANOVA) followed by Tukey’s post hoc analysis were applied. *P*<0.05 was considered statistically significant.
